# *Withania somnifera* modulates cancer cachexia associated inflammatory cytokines and cell death in leukaemic THP-1 cells and peripheral blood mononuclear cells (PBMC’s)

**DOI:** 10.1186/s12906-018-2192-y

**Published:** 2018-04-10

**Authors:** Dhaneshree Bestinee Naidoo, Anil Amichund Chuturgoon, Alisa Phulukdaree, Kanive Parashiva Guruprasad, Kapaettu Satyamoorthy, Vikash Sewram

**Affiliations:** 1 Discipline of Medical Biochemistry, Faculty of Health Sciences, Nelson Mandela School of Medicine, University of KwaZulu-Natal, Durban, 4013 South Africa; 2 Division of Biotechnology, School of Life Sciences, Manipal University, Planetarium Complex, Manipal, Karnataka 576 104 India; 3 African Cancer Institute, Stellenbosch University, P.O. Box 241, Cape Town, 8000 South Africa; 4 Department of Global Health, Faculty of Medicine and Health Sciences, Stellenbosch University, P.O. Box 241, Cape Town, 8000 South Africa

**Keywords:** Cancer, Cachexia, Cytokines, Apoptosis, *Withania somnifera*

## Abstract

**Background:**

Cancer and inflammation are associated with cachexia. *Withania somnifera* (*W. somnifera*) possesses antioxidant and anti-inflammatory potential. We investigated the potential of an aqueous extract of the root of *W. somnifera* (W_RE_) to modulate cytokines, antioxidants and apoptosis in leukaemic THP-1 cells and peripheral blood mononuclear cells (PBMC’s).

**Methods:**

Cytotoxcity of W_RE_ was determined at 24 and 72 h (h). Oxidant scavenging activity of W_RE_ was evaluated (2, 2-diphenyl-1 picrylhydrazyl assay). Glutathione (GSH) levels, caspase (− 8, − 9, − 3/7) activities and adenosine triphosphate (ATP) levels (Luminometry) were thereafter assayed. Tumour necrosis factor-α (TNF-α), interleukin (IL)-6, IL-1β and IL-10 levels were also assessed using enzyme-linked immunosorbant assay.

**Results:**

At 24 h, W_RE_ (0.2–0.4 mg/ml) decreased PBMC viability between 20 and 25%, whereas it increased THP-1 viability between 15 and 23% (*p* < 0.001). At 72 h, W_RE_ increased PBMC viability by 27–39% (0.05, 0.4 mg/ml W_RE_) whereas decreased THP-1 viability between 9 and 16% (0.05–0.4 mg/ml W_RE_) (*p* < 0.001). Oxidant scavenging activity was increased by W_RE_ (0.05–0.4 mg/ml, *p* < 0.0001). PBMC TNF-α and IL-10 levels were decreased by 0.2–0.4 mg/ml W_RE,_ whereas IL-1β levels were increased by 0.05–0.4 mg/ml W_RE_ (*p* < 0.0001). In THP-1 cells, W_RE_ (0.05–0.4 mg/ml) decreased TNF-α, IL-1β and IL-6 levels (*p* < 0.0001). At 24 h, GSH levels were decreased in PBMC’s, whilst increased in THP-1 cells by 0.2–0.4 mg/ml W_RE_ (*p* < 0.0001). At 72 h, W_RE_ (0.1–0.4 mg/ml) decreased GSH levels in both cell lines (*p* < 0.0001). At 24 h, W_RE_ (0.2–0.4 mg/ml) increased PBMC caspase (-8, -3/7) activities whereas W_RE_ (0.05, 0.1, 0.4 mg/ml) increased THP-1 caspase (-9, -3/7) activities (*p* < 0.0001). At 72 h, PBMC caspase (-8, -9, -3/7) activities were increased at 0.05–0.1 mg/ml W_RE_ (*p* < 0.0001). In THP-1 cells, caspase (-8, -9, -3/7) activities and ATP levels were increased by 0.1–0.2 mg/ml W_RE,_ whereas decreased by 0.05 and 0.4 mg/ml W_RE_ (72 h, *p* < 0.0001).

**Conclusion:**

In PBMC’s and THP-1 cells, W_RE_ proved to effectively modulate antioxidant activity, inflammatory cytokines and cell death. In THP-1 cells, W_RE_ decreased pro-inflammatory cytokine levels, which may alleviate cancer cachexia and excessive leukaemic cell growth.

**Electronic supplementary material:**

The online version of this article (10.1186/s12906-018-2192-y) contains supplementary material, which is available to authorized users.

## Background

Chronic inflammation plays an essential role in malignancies [1] through the initiation, promotion and progression of tumours [2]. Usually, the host-mediated anti-tumour activity overcomes the tumour-mediated immunosuppressive activity leading to the elimination of cancerous cells [2]. However, in the presence of an inadequate host anti-tumour defence, the pro-inflammatory tumour microenvironment is enhanced and promotes tumour development, invasion, angiogenesis and metastasis [2].

The cachectic syndrome is prominent in malignancies occurring in up to 50% of all cancer patients [3]. It is a progressive, debilitating condition leading to abnormal weight loss, as a result of adipose tissue (85%) and skeletal muscle (75%) depletion [3–5]. Modulation of lipogenesis and lipolysis is essential in maintaining adipose tissue mass. Lipoprotein lipase (LPL) hydrolyses fatty acids (FA’s) from plasma lipoproteins, thereafter FA’s are transported to adipose tissue for triacylglycerol (TAG) production, whereas hormone sensitive lipase (HSL) hydrolyses TAG’s into FA’s and glycerol [3]. Literature shows that decreased serum LPL levels/activity and increased HSL levels/activity are associated with cachexia [6–8]. Additionally, increased proteolysis [9] and decreased proteogenesis has been established in cachectic patients [10]. The ATP-ubiquitin-dependent proteolytic pathway has been shown to be responsible for the accelerated proteolysis seen in a variety of wasting conditions, including cancer cachexia [11].

Inflammatory cytokines, oxidative stress and apoptosis have been implicated in the initiation and progression of cancer, imbalance of catabolic/anabolic processes [12] and development of cachexia [13]. Production of inflammatory cytokines [tumour necrosis factor-α (TNF-α), interleukin (IL) – 6, and IL-1β] is activated by lipopolysaccharide (LPS) that potently stimulates macrophages [14, 15]. The LPS signal is transduced by LPS binding to LPS binding protein, delivered to CD14 and transferred to Toll like receptor-4 [16]. Nuclear factor kappa B (NF-κB) is subsequently activated and regulates the transcription of genes associated with inflammation, proliferation, invasion, angiogenesis and apoptosis [1, 17–19]. Pro-inflammatory cytokines (TNF-α, IL-6 and IL-1) have been shown to decrease LPL activity [20–23], which reduces the uptake of exogenous lipids by adipose tissue [21], ultimately decreasing lipogenesis. Additionally, previous studies have indicated that TNF-α increased ubiquitin (concentrations and mRNA), while IL-6 increased the 26S proteasome and cathepsins activities, which may activate proteolytic pathways [4, 24–26], ultimately increasing proteolysis. In combination, excessive levels of pro-inflammatory cytokines increase tumour suppressive activity [2] and tissue wasting [3].

Reactive oxygen species (ROS) have been associated with tumour initiation, inflammation [2, 27] and muscle wasting [28]. However, antioxidants have been shown to decrease muscle wasting by neutralizing ROS [1, 28]. Elevated ROS levels activate apoptotic pathways, ultimately activating caspase-3 [29]. Caspase-3 activation plays an essential role in the execution of apoptosis, as well as muscle proteolysis [30]. In addition, in weight losing upper gastro-intestinal tract cancer patients, deoxyribonucleic acid (DNA) fragmentation and poly (ADP-ribose) polymerase (PARP) cleavage were increased, whereas MyoD protein was decreased, indicating increased apoptosis and decreased muscle replenishment [3].

Cancer patients suffer from a wide range of side-effects caused by current cancer chemotherapeutic and radiotherapeutic agents. Patients are constantly seeking alternative traditional remedies to alleviate their discomfort. *Withania somnifera* (L.) Dunal (*W. somnifera*) is a well known medicinal plant cultivated in India, parts of East Asia and Africa [31]. It is commonly referred to as Ashwagandha and belongs to the Solanaceae family [31]. Compounds isolated from *W. somnifera* include withaferin A and 3-β-hydroxy-2, 3 dihydro withanolide F [32]. The major constituent of the root extract of *W. somnifera* is withanolide-A [33]. *W. somnifera* is frequently used in Ayurvedic medicine due to its various medicinal properties [31]. These properties include anti-inflammatory [34], antioxidant and immune-modulatory activities [35]. *W. somnifera* was found to be an immune regulator in inflammation animal models [36]. The immunosuppressive action of *W. somnifera* may be due to the presence of withanolides, steroidal lactones and a few flavanoids [37]. In addition, *W. somnifera* formulation (WSF) has shown anti-proliferative potential in human promyelocytic leukemia (HL-60) cells, by activating the intrinsic and extrinsic apoptotic pathways [38]. When used together, *W. somnifera* formulations aid the host to effectively fight cancer and reduce the harmful effects of chemotherapy and radiotherapy [39].

There is a need for the discovery of an inexpensive cancer cachectic treatment to improve the prognosis of cancer patients and to establish a mechanism of regulation of the immune system, inflammasome and apoptosis in order to prevent/decelerate the rapid depletion of skeletal muscle and adipose tissue. We investigated the effect of an aqueous extract of the root of *W. somnifera* (W_RE_) on antioxidant capacity, inflammatory cytokine levels and cell death induction in leukaemic THP-1 cells and peripheral blood mononuclear cells (PBMC’s).

## Methods

### Materials

The roots of *W. somnifera* were collected on the 11th of March 2011 (collectors number: Immelman 427) from the Eastern Cape [the Ntubeni Location near Dwesa Reserve], South Africa (SA) and identified by Dr. Kathleen Immelman from the Department of Botany at the Walter Sisulu University, SA and further comparison to South African data [40]. Voucher specimens were deposited at the KEI herbarium (13995). THP-1 cells were obtained from from American Type Culture Collection (ATCC, University Boulevard Manassas, Virginia, USA). RPMI-1640 and BD OptEIA enzyme-linked immunosorbant assay (ELISA) cytokine kits were purchased from The Scientific Group (Johannesburg, SA). Foetal calf serum (FCS) and Pen/Strep Amphotericin B (PSF) were acquired from Whitehead Scientific (Cape Town, SA). Dimethyl sulphoxide (DMSO) was purchased from Merck (Johannesburg, SA). Histopaque-1077, LPS and 2, 2-diphenyl-1 picrylhydrazyl (DPPH) were purchased from Sigma (Aston Manor, SA). The 4-[3-(4-iodophenyl)-2-(4-nitrophenyl)-2H-5-tetrazolio]-1,3-benzene disulphonate (WST-1) cell proliferation reagent was purchased from Roche (Johannesburg, SA). Promega (Madison, USA) supplied the caspase (-3/7, -8, -9), adenosine triphosphate (ATP) and glutathione (GSH) kits.

### Plant extraction

The roots of *W. somnifera* were dried and milled before being sequentially extracted in ethanol and distilled water. Ethanol (200–350 ml) was added to the milled root (10–30 g) and extracted overnight by shaking (4×g, 37 °C). The ethanol extracts were thereafter filtered, evaporated using a rotary evaporator, dried (37 °C) and stored (4 °C). The root material was thereafter extracted with distilled water (200–350 ml) by shaking (4×g, 75 °C) for a period of 6 hours (h). Water extracts were filtered, dried and stored (4 °C).

### The 2, 2-diphenyl-1 picrylhydrazyl assay

W_RE_ (0.05–0.4 mg/ml) and butylated hydroxytoluene (BHT) (60–300 μM) dilutions were prepared in methanol (99.5% and grade AR). A 50 μM DPPH solution was prepared from a stock solution of 0.135 mM DPPH in methanol. W_RE_, BHT dilutions and methanol (1 ml) were aliquoted into 15 ml polypropylene tubes, followed by the 50 μM DPPH solution (1 ml). Reaction mixtures were vortexed and incubated [room temperature (RT), 30 min (min)] in the dark. Absorbance of samples was read at 517 nm using a Varine Cary 50 UV-visible spectrophotometer (McKinley Scientific, New Jersey, US).

### Isolation of peripheral blood mononuclear cells

Buffy coats containing PBMC’s were obtained from the South African National Blood Service (2011/09). PBMC’s were extracted by differential centrifugation. Buffy coats (5 ml) were layered onto equivolume histopaque-1077 (5 ml) in 15 ml polypropylene tubes and centrifuged (400×g, 21 °C for 30 min). After centrifugation, the PBMC’s were transferred to sterile 15 ml polypropylene tubes, phosphate buffered saline (PBS) was added (0.1 M, 10 ml) and tubes were centrifuged (400×g, 21 °C, 15 min). Cell density of isolated PBMC’s was adjusted (1 × 10^6^ cells/ml) using the trypan blue exclusion test and cryo-preserved (10% FCS, 10% DMSO) using a NELGENE cryo freezing container and stored at -80 °C.

### Tissue culture

THP-1 cells were grown in the appropriate tissue culture conditions in a 75 cm^3^ tissue culture flask (37 °C, 5% CO_2_). The growth media comprised of RPMI-1640, FCS (10%) and PSF (2%). Cells were thawed, seeded into a 75 cm^3^ tissue culture flask at a concentration of 3 × 10^5^ cells/ml and incubated (37 °C, 5% CO_2_). THP-1 cells were allowed to grow for 2–3 days before the cells were centrifuged (162×g, 10 min) and re-suspended in fresh growth media. The number of cells should not exceed 8 × 10^5^ cells/ml, therefore the cells/ml was quantified daily by trypan blue staining. Once the cell count reached 8 × 10^5^ cells/ml the THP-1 cells were split/diluted to 3 × 10^5^ cells/ml with media and incubated. Subsequent experiments were conducted once the cell numbers were sufficient.

### Cell viability assay

Cytotoxicity of W_RE_ in PBMC’s and THP-1 cells was measured using the WST-1 assay (Roche, Johannesburg, SA). PBMC and THP-1 cells (10,000 cells/well, 96-well plate, in triplicate wells) were stimulated with LPS (20 μg/ml, 37 °C, 5% CO_2_, 4 h) before exposure to W_RE_ (0.05–0.4 mg/ml) for 24 and 72 h (37 °C, 5% CO_2_). Similarly, controls received media. Thereafter, plates were centrifuged (162×g, 10 min), supernatant removed, cell pellets re-suspended in growth media (100 μl/well), WST-1 reagent (10 μl/well) added and plates incubated (37 °C, 5%, CO_2_, 3 h). Optical density was measured at 450 nm (620 nm reference wavelength) with a BIO-TEK μQuant spectrophotometer (Analytical and Diagnostic Products, SA). This experiment was conducted independently on three occasions.

### Stimulation and treatment of cells

PBMC’s and THP-1 cells (1 × 10^5^ cells/ml) were transferred into 24-well plates and stimulated with LPS (20 μg/ml, 37 °C, 5% CO_2_, 4 h) before exposure to W_RE_ (0.05–0.4 mg/ml) for 24 h (TNF-α) and 72 h (IL-1β, IL-6, IL-10) (37 °C, 5% CO_2_). After incubation, plates were centrifuged (162×g, 10 min) and the supernatant collected and stored (− 80 °C) for cytokine analysis. Cell pellets were used to conduct the caspase (-8, -9, -3/7) activity, as well as ATP and GSH assays. The experiments were conducted independently (twice) for all subsequent assays.

### Quantification of cytokines

Cytokine levels were estimated using the BD OptEIA ELISA kits (The Scientific Group, SA) and the procedure was followed as per the instruction manual. ELISA plates were coated with capture antibody overnight (100 μl/well, 4 °C). Thereafter, plates were washed (3×) with wash buffer and blocked with assay diluent (200 μl/well, 1 h, RT). Standard solutions were prepared by diluting a stock solution [TNF-α, IL-10 (500 pg/ml), IL-6 (300 pg/ml), IL-1β (250 pg/ml)] serially [TNF-α, IL-10 (500–7.8 pg/ml), IL-6 (300–4.7 pg/ml), IL-1β (250–3.9 pg/ml)]. Plates were washed (3×), standards and samples (100 μl/well) were aliquoted into appropriate wells and plates were incubated (2 h, RT). Plates were washed (5×), working detector (100 μl/well) added and plates incubated (1 h, RT). The plates were washed (7×), substrate solution (100 μl/well) added and plates were incubated (30 min, RT) in the dark. Finally, stop solution (50 μl/well) was added and the absorbance was read at 450 nm (570 nm reference wavelength) with a Multiskan FC micro-plate reader (Thermo Scientific). Cytokine concentrations were calculated by extrapolation from a standard curve.

### The glutathione assay

The GSH-Glo™ assay (Promega, Madison, USA) was performed to measure GSH levels. Standard GSH solutions were prepared by diluting a 5 mM stock solution serially (1.56–50 μM) and PBS (0.1 M) was the standard blank. Cells (50 μl/well, 2 × 10^5^ cells/ml) and standards were added into an opaque 96-well plate, followed by GSH-Glo™ reagent (25 μl/well) and allowed to incubate (30 min, RT) in the dark. Luciferin detection reagent (50 μl/well) was subsequently added and plates incubated (15 min, RT) in the dark. The absorbance was read on a Modulus™ microplate luminometer (Turner Biosystems, Sunnyvale, USA) and GSH concentrations were calculated by extrapolation from a standard curve.

### Caspase and ATP assays

Caspase activity and ATP levels were determined using the Caspase-Glo®-3/7, -8, -9 and ATP assay kits (Promega, Madison, USA). Caspase-Glo®-3/7, -8, -9 and ATP reagents were reconstituted according to the manufacturer’s instructions. Cells (100 μl, 2 × 10^5^ cells/ml) were added into duplicate wells of a microtitre plate for each assay, thereafter caspase -3/7, -8, -9 and ATP reagents (100 μl/well) were added into appropriate wells. The plate was incubated (30 min, RT) in the dark. Luminescence was measured on a Modulus™ microplate luminometer (Turner BioSystems) and expressed as relative light units (RLU).

### Statistical analysis

Statistical analysis was performed using the STATA and GraphPad Prism statistical analysis software. The one-way analysis of variance (ANOVA) was used to compare between groups, followed by the Tukey multiple comparisons test, with *p* < 0.05 defining statistical significance.

## Results

### The oxidant scavenging potential of W_RE_

**Fig. 1:**
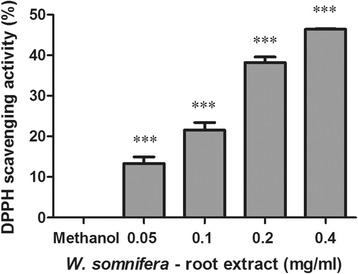
Percentage DPPH scavenging activity of W_RE_ (Values expressed as mean ± SD, ^*^ *p* < 0.05, ^***^ *p* < 0.0001, compared to control)

The oxidant scavenging activity of W_RE_ using the DPPH assay is shown in Fig. 1. W_RE_ (0.05–0.4 mg/ml) significantly increased DPPH scavenging activity by 13.33–46.38% (Fig. 1, *p* < 0.0001).

### The in vitro cytotoxicity of W_RE_

**Fig. 2:**
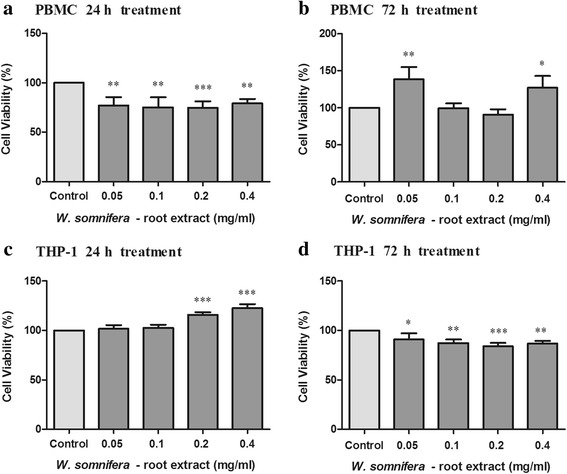
Cell viability of PBMC’s (**a** – 24 h, **b** – 72 h) and THP-1 (**c** – 24 h, **d** – 72 h) cells treated with W_RE_ for 24 and 72 h (Values expressed as mean ± SD, ^*^ *p* < 0.05,^**^ *p* < 0.005, ^***^ *p* < 0.0001 compared to the control)

The WST-1 assay was used to determine cell viability of THP-1 cells and PBMC’s after treatment with W_RE_ (Fig. 2). At 24 h, W_RE_ (0.05–0.4 mg/ml) decreased PBMC viability by 20.69–25.15% while W_RE_ (0.2–0.4 mg/ml) increased THP-1 viability by 15.99–22.54% as compared to the controls (Fig. 2a and c, *p* < 0.001). This result suggests that PBMC’s are more sensitive to W_RE_ treatment than THP-1 cells.

At 72 h, PBMC viability was increased (27.16–38.58%) by W_RE_ (0.05, 0.4 mg/ml)_,_ as compared to the control (Fig. 2b, *p* < 0.0001). In the same time period, W_RE_ (0.05–0.4 mg/ml) decreased THP-1 viability by 9.07–16.09% relative to the control (Fig. 2d, *p*=0.0002).

### The immune suppressive properties of W_RE_

**Fig. 3:**
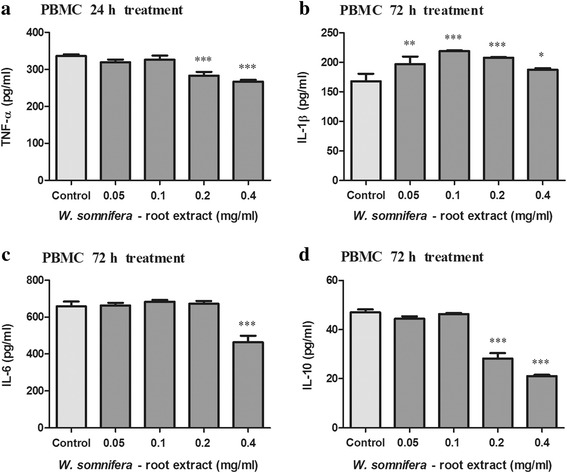
Concentration of TNF-α (**a**), IL-1β (**b**), IL-6 (**c**) and IL-10 (**d**) in LPS stimulated and W_RE_ treated PBMC’s (Values expressed as mean ± SD, ^*^p < 0.05, ^***^ *p* < 0.0001, compared to the control)

**Fig. 4:**
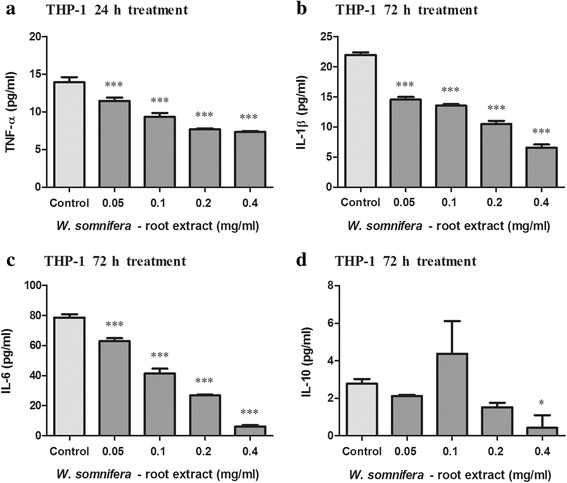
Concentration of TNF-α (**a**), IL-1β (**b**), IL-6 (**c**) and IL-10 (**d**) in LPS stimulated and W_RE_ treated THP-1 cells (Values expressed as mean ± SD, ^*^p < 0.05, ^***^p < 0.0001 compared to the control)

W_RE_ altered cytokine levels in PBMC’s and THP-1 cells (Fig. 3 and Fig. 4). The levels of TNF-α, IL-1β, IL-6 and IL-10 produced by LPS stimulated PBMC’s was 336.218, 168.100, 657.878 and 46.990 pg/ml respectively. W_RE_ (0.2–0.4 mg/ml) decreased PBMC TNF-α and IL-10 levels as compared to the control (Fig. 3a and d, *p* < 0.0001). In PBMC’s, IL-6 levels were decreased by 0.4 mg/ml W_RE,_ whereas IL-1β levels were increased by 0.05–0.4 mg/ml W_RE_ relative to the control (Fig. 3b and c, *p* < 0.0001).

The levels of TNF-α, IL-1β, IL-6 and IL-10 produced by LPS stimulated THP-1 cells were 13.285, 21.947, 78.622 and 2.705 pg/ml respectively. In THP-1 cells, TNF-α, IL-1β and IL-6 levels were decreased by 0.05–0.4 mg/ml W_RE,_ whilst IL-10 levels were decreased by 0.4 mg/ml W_RE_ as compared to the control (Fig. 4, *p* < 0.003).

### The antioxidant potential of W_RE_

The endogenous antioxidant activity of W_RE_ was determined by measuring GSH levels in both cell lines (Table 1). At 24 h, GSH levels in PBMC’s were decreased by W_RE_ (0.05, 0.2, 0.4 mg/ml) relative to the control (Table 1, *p* < 0.0001). In THP-1 cells, GSH levels were decreased at 0.05 mg/ml W_RE_ whereas increased (0.41–1.62 μM) at 0.1–0.4 mg/ml W_RE_ compared to the control (Tables 1, 24 h, *p* < 0.0001).

**Table 1:** Glutathione levels in LPS stimulated and W_RE_ treated PBMC’s and THP-1 cells

Glutathione (μM)				
W_RE_ (mg/ml)	24 h Treatment		72 h Treatment	
	PBMC	THP-1	PBMC	THP-1
Control	1.613 ± 0.017	1.632 ± 0.004	4.799 ± 0.008	1.608 ± 0.004
0.05	1.442 ± 0.024 ^***^	1.267 ± 0.004 ^***^	5.232 ± 0.011 ^***^	1.548 ± 0.002 ^***^
0.1	1.617 ± 0.002	2.045 ± 0.002 ^***^	4.015 ± 0.001 ^***^	1.589 ± 0.004 ^***^
0.2	1.390 ± 0.001 ^***^	3.253 ± 0.017 ^***^	2.323 ± 0.005 ^***^	1.401 ± 0.006 ^***^
0.4	1.321 ± 0.006 ^***^	2.785 ± 0.005 ^***^	4.697 ± 0.003 ^***^	1.411 ± 0.005 ^***^

(Values expressed as mean ± SD, ^***^ *p* < 0.0001, compared to the control)

At 72 h, PBMC GSH levels were increased at 0.05 mg/ml whereas decreased 0.1–0.4 mg/ml W_RE_ compared to the control (Table 1, *p* < 0.0001). W_RE_ (0.05–0.4 mg/ml) decreased GSH levels in THP-1 cells relative to the control (Tables 1, 72 h *p* < 0.0001).

### W_RE_ modulates caspase (-8, -9, -3/7) activities and ATP levels

Luminometry assays were used to determine caspase activity and ATP levels in THP-1 cells and PBMC’s after treatment with W_RE_. The pro-apoptotic effect of W_RE_ in PBMC’s treated for 24 h is shown in Table 2. At 24 h, PBMC caspase-8 activity was decreased by 0.05 mg/ml W_RE_ whereas increased by 0.2–0.4 mg/ml W_RE_ compared to the control (Table 2, *p* < 0.0001). PBMC caspase-9 activity was increased by 0.05 and 0.2 mg/ml W_RE_ but decreased by 0.1 and 0.4 mg/ml W_RE_ relative to the control (Table 2, *p* < 0.0001). In PBMC’s, the increased caspase activity may be related to the decreased GSH levels at 24 h. A decrease in GSH levels may allow for an increase in ROS levels which can activate apoptotic pathways. Caspase-3/7 activity was increased in PBMC’s by 0.05–0.4 mg/ml W_RE_ compared to the control (Table 2, *p* < 0.0001), suggesting an increased execution of apoptotic cell death. In azoxymethane-induced colon cancer in mice, *W. somnifera* has been shown to modulate TCA cycle enzymes and the electron transport chain [41]. The PBMC ATP levels were increased by 0.1, 0.4 mg/ml W_RE_ but decreased by 0.05, 0.2 mg/ml W_RE_ compared to the control (Table 2, *p* < 0.0001), which may be related to the modulation of the electron transport chain by *W. somnifera*.

**Table 2:** Modulation of caspase (-8, -9, -3/7) activity and ATP levels in LPS stimulated and 24 h W_RE_ treated PBMC’s

W_RE_ (mg/ml)	Caspase-8 (RLU × 10^5^)	Caspase-9 (RLU × 10^5^)	Caspase-3/7 (RLU × 10^5^)	ATP (RLU × 10^5^)
Control	0.185 ± 0.006	0.366 ± 0.0003	7.756 ± 0.006	4.714 ± 0.004
0.05	0.155 ± 0.0002 ^***^	0.376 ± 0.001 ^***^	8.109 ± 0.094 ^**^	2.783 ± 0.017 ^***^
0.1	0.192 ± 0.00002	0.253 ± 0.0002 ^***^	11.504 ± 0.253 ^***^	5.208 ± 0.005 ^***^
0.2	0.246 ± 0.0003 ^***^	0.397 ± 0.0005 ^***^	8.961 ± 0.015 ^***^	3.741 ± 0.033 ^***^
0.4	4.814 ± 0.006 ^***^	0.351 ± 0.001 ^***^	17.095 ± 0.089 ^***^	6.965 ± 0.039 ^***^

(Values expressed as mean ± SD, ^**^ *p* < 0.005, ^***^ *p* < 0.0001 compared to the control)

W_RE_ pro-apoptotic effects in THP-1 cells treated for 24 h are shown in Table 3. At 24 h, THP-1 caspase-9 activity was decreased by 0.2 mg/ml W_RE_ but increased by 0.05, 0.1, 0.4 mg/ml W_RE_ compared to the control (Table 3, *p* < 0.0001). At 0.2 mg/ml W_RE_, the decreased caspase-9 activity may be related to the increased GSH levels. An increase in GSH levels may decrease ROS levels thus minimising mitochondrial depolarisation and the activation of the intrinsic apoptotic pathway. In THP-1 cells, W_RE_ (0.05–0.4 mg/ml) decreased caspase-8 activity, whereas increased caspase-3/7 activity and ATP levels relative to the control (Table 3, *p* < 0.0001). Elevated caspase (-9, -3/7) activities suggests the initiation of the mitochondrial apoptotic pathway.

**Table 3:** Modulation of caspase (-8, -9, -3/7) activity and ATP levels in LPS stimulated and 24 h W_RE_ treated THP-1 cells

W_RE_ (mg/ml)	Caspase-8 (RLU × 10^5^)	Caspase-9 (RLU × 10^5^)	Caspase-3/7 (RLU × 10^5^)	ATP (RLU × 10^5^)
Control	10.207 ± 0.011	1.040 ± 0.007	1.251 ± 0.016	2.636 ± 0.011
0.05	8.440 ± 0.039 ^***^	2.365 ± 0.005 ^***^	1.315 ± 0.005 ^***^	3.726 ± 0.005 ^***^
0.1	2.413 ± 0.005 ^***^	2.459 ± 0.002 ^***^	2.294 ± 0.006 ^***^	5.132 ± 0.014 ^***^
0.2	7.149 ± 0.027 ^***^	0.775 ± 0.002 ^***^	3.406 ± 0.006 ^***^	29.838 ± 0.186 ^***^
0.4	2.456 ± 0.033 ^***^	3.197 ± 0.0001 ^***^	9.428 ± 0.004 ^***^	10.282 ± 0.195 ^***^

(Values expressed as mean ± SD, ^***^ *p* < 0.0001 compared to the control)

The pro-apoptotic effect of W_RE_ in PBMC’s treated for 72 h is shown in Table 4. At 72 h, PBMC caspase-8 activity was increased by 0.05–0.2 mg/ml W_RE_ but decreased by 0.4 mg/ml W_RE_ compared to the control (Table 4, *p* < 0.0001). PBMC caspase-9 activity was increased by 0.05–0.1 mg/ml W_RE_ but decreased by 0.2–0.4 mg/ml W_RE_ relative to the control (Table 4, *p* < 0.0001). In PBMC’s, caspase-3/7 activity was increased by 0.05, 0.1, 0.4 mg/ml W_RE_ whereas it decreased by 0.2 mg/ml W_RE_ compared to the control (Table 4, *p* < 0.0001). At 0.05–0.1 mg/ml W_RE_, the increased caspase-3/7 activity is consistent with the significantly increased caspase -8 and -9 activity. At 0.2 mg/ml W_RE_, caspase-8 activity was minimally increased and caspase-9 activity significantly decreased which lead to the decreased caspase-3/7 activity. At 0.4 mg/ml W_RE_, although both caspase -8 and -9 activities were decreased, caspase-3/7 activity was increased. A previous study has demonstrated that one activated executioner caspase can cleave and activate other executioner caspases resulting in positive feedback loop of caspase activation [42] which may account for the increased caspase-3/7 activity at 0.4 mg/ml W_RE_. W_RE_ (0.05–0.4 mg/ml) decreased PBMC ATP levels relative to the control (Table 4, *p* < 0.0001).

**Table 4:** Modulation of caspase (-8, -9, -3/7) activity and ATP levels in LPS stimulated and 72 h W_RE_ treated PBMC’s

W_RE_ (mg/ml)	Caspase-8 (RLU × 10^5^)	Caspase-9 (RLU × 10^5^)	Caspase-3/7 (RLU × 10^5^)	ATP (RLU × 10^5^)
Control	42.651 ± 0.039	115.041 ± 3.848	155.556 ± 0.387	20.574 ± 0.316
0.05	53.840 ± 0.026 ^***^	143.861 ± 3.929 ^***^	196.471 ± 0.338 ^***^	10.223 ± 0.046 ^***^
0.1	52.109 ± 0.009 ^***^	129.033 ± 0.289 ^***^	192.695 ± 0.233 ^***^	12.506 ± 0.373 ^***^
0.2	42.751 ± 0.039 ^**^	105.494 ± 4.247 ^**^	154.203 ± 0.224 ^***^	13.210 ± 0.043 ^***^
0.4	29.656 ± 0.007 ^***^	92.718 ± 0.021 ^***^	165.139 ± 0.096 ^***^	13.361 ± 0.279 ^***^

(Values expressed as mean ± SD,^**^ *p* < 0.005, ^***^ *p* < 0.0001 compared to the control)

W_RE_ pro-apoptotic effects in THP-1 cells treated for 72 h are shown in Table 5. At 72 h, THP-1 caspase (-8, -9, -3/7) activity and ATP levels were increased by 0.1–0.2 mg/ml W_RE_ as compared to the control (Table 5, *p* < 0.0001), suggesting an increase in THP-1 apoptotic cell death. THP-1 caspase (-8, -9, -3/7) activity and ATP levels were decreased by 0.05, 0.4 mg/ml W_RE_ relative to the control (Table 5, 72 h, *p* < 0.0001), suggesting a decrease in THP-1 apoptosis.

**Table 5:** Modulation of caspase (-8, -9, -3/7) activity and ATP levels in LPS-stimulated and 72 h W_RE_-treated THP-1 cells

W_RE_ (mg/ml)	Caspase-8 (RLU × 10^5^)	Caspase-9 (RLU × 10^5^)	Caspase-3/7 (RLU × 10^5^)	ATP (RLU × 10^5^)
Control	0.991 ± 0.0001	5.738 ± 0.002	7.463 ± 0.012	4.332 ± 0.002
0.05	0.978 ± 0.0001 ^***^	5.562 ± 0.009 ^***^	6.919 ± 0.003 ^***^	4.133 ± 0.005 ^***^
0.1	1.216 ± 0.001 ^***^	7.045 ± 0.005 ^***^	8.211 ± 0.002 ^***^	4.889 ± 0.005 ^***^
0.2	1.095 ± 0.001 ^***^	6.091 ± 0.001 ^***^	7.532 ± 0.006 ^***^	4.576 ± 0.004 ^***^
0.4	0.952 ± 0.0003 ^***^	5.639 ± 0.003 ^***^	6.626 ± 0.007 ^***^	4.039 ± 0.0003 ^***^

(Values expressed as mean ± SD, ^***^ *p* < 0.0001 compared to the control)

## Discussion

Cachexia patients experience excessive weight loss due to increased lipolysis and proteolysis which have been linked to elevated levels of pro-inflammatory cytokines, oxidative stress and apoptosis [3, 5, 30]. Previously, the powdered root of *W. somnifera* displayed immune modulatory properties [43] and WSF has been shown to increase caspase-3 activity, subsequently inducing apoptosis [38]. The objective of this study was thus to investigate the modulation of cytokines, antioxidants and cell death by W_RE_ in PBMC’s and THP-1 cells.

Dhanani et al. (2017) showed that the root extract of *W. somnifera* inhibited 50% of DPPH at a concentration of 0.4 mg/ml [44]. Our results indicated that W_RE_ has oxidant scavenging potential ranging between 13 and 46% at 0.05–0.4 mg/ml. ROS plays an essential role in tumour initiation, inflammation, protein degradation and apoptosis. The antioxidant potential of W_RE_ may decrease inflammatory cytokine levels as well as ROS induced apoptosis.

At 24 h, the WST-1 results indicated that W_RE_ decreased PBMC viability whilst increasing THP-1 viability. However at 72 h, W_RE_ increased PBMC viability whilst conversely decreasing THP-1 viability. In contrast, the growth of various cell lines (HT-29, HCT-15, SW620, 502,713, Colo-205, A549, HOP-62 and Hep-G2) were dose dependently inhibited by WSF and 50% cell growth inhibition was seen at 30 μg/ml WSF [38].

The pivotal role of inflammatory cytokines in malignancies and cachexia has been extensively documented [3]. Dhuley (1997) previously reported that *W. somnifera* inhibits macrophage production of inflammatory cytokines (IL-1, TNF-α) [45]. Our results showed that W_RE_ decreased PBMC TNF-α, IL-10 and IL-6 levels, although it increased IL-1β levels. In THP-1 cells, pro-inflammatory cytokine (TNF-α, IL-1β, IL-6) levels were significantly decreased by W_RE_.

Pro-inflammatory cytokines, over a longstanding time period, stimulate the production of genotoxic molecules [nitric oxide (NO), ROS] and tumour progression by promoting angiogenesis and metastasis [1, 2]. In addition, pro-inflammatory cytokines activate NF-κB which regulates the expression of genes involved in the suppression of tumour apoptosis, stimulation of tumour cell cycle progression and enhancement of inflammatory mediators [1, 2]. NF-κB promotes tumour progression, invasion, angiogenesis and metastasis [1, 2].

Previous literature has shown that IL-1 stimulates growth and invasion of malignant cells [2]. Additionally, IL-6 has been shown to target cell cycle progression and anti-apoptotic genes leading to tumour proliferation and anti-apoptotic potential [2]. The ability of W_RE_ to increase pro-inflammatory cytokines such as IL-1β in PBMC’s may aid in cancerous cell elimination through increased host anti-tumour activity. Conversely, in THP-1 cells, the decrease in TNF-α, IL-6 and IL-1β levels by W_RE_ may prevent excessive activation of NF-κB, diminish cytokine induced tumour immunosuppressive activity and cancer progression.

With regard to cancer cachexia, IL-6 decreased LPL activity in adipose tissue of mice [22] and IL-1 directly modulates lipid metabolism by suppressing LPL activity [23]. TNF-α decreased LPL activity in adipose tissue of human (maintained in organ culture), rat, mouse, and guinea pigs [21]. Additionally, TNF-α inhibits the production of LPL and reduces the rate of LPL gene transcription in mouse 3 T3-L1 adipocytes, hence preventing the formation of new lipid stores while stimulating HSL and increasing lipolysis [3, 20, 46]. The potential of W_RE_ to decrease pro-inflammatory cytokine levels in PBMC’s and THP-1 cells suggests a decrease in LPL inhibition and HSL stimulation, thus maintaining lipogenesis and minimizing lipolysis. IL-6 and TNF-α further contribute to cachexia by stimulating muscle catabolism via the activation of proteasome pathways [24, 25, 47]. In cachexia, NF-κB activation induces ubiquitin–proteasome pathway activity and suppresses MyoD expression [48], thereby increasing proteolysis and reducing muscle replenishment [49]. By decreasing TNF-α and IL-6 levels in PBMC’s and THP-1 cells, W_RE_ may prevent excessive activation of NF-κB and proteasome pathways, ultimately decreasing proteolysis associated with the cachectic syndrome. Taken together, W_RE_ may be able to decrease tissue wasting through the down regulation of pro-inflammatory cytokine levels.

The immunosuppressive and anti-inflammatory cytokine, IL-10, inhibits tumour development, tumour progression, modulates apoptosis and suppresses angiogenesis during tumour regression [1, 2]. Additionally, IL-10 inhibits NF-κB activation and subsequently inhibits pro-inflammatory cytokine production (TNF-α, and IL-6) [2]. In PBMC’s and THP-1 cells, the decreased IL-10 levels may be due to IL-10 combating increased pro-inflammatory cytokines levels (TNF-α, IL-6, IL-1β).

Antioxidants protect cells from increased oxidative stress [50]. GSH is a potent antioxidant that effectively scavenges ROS both directly and indirectly [50]. *W. somnifera* has previously been shown to possess chemo-preventive activity which may be a consequence of its antioxidant capacity [39]. The 24 h results showed that W_RE_ decreased GSH levels in PBMC’s, whereas it increased GSH levels in THP-1 cells. However, at 72 h, W_RE_ decreased GSH levels in both cell lines. Notably, GSH levels (72 h) were higher in control PBMC’s (4.79 μM) compared to control THP-1 cells (1.61 μM), suggesting a higher oxidant defence in PBMC’s.

The extrinsic (death receptors) and intrinsic (mitochondria) pathways are the two main apoptotic pathways [29]. Activation of initiator caspases (-8, -9) leads to the activation of executioner caspases (-3/7) resulting in activation of cytoplasmic endonucleases [29]. In HL-60 cells, WSF treatment led to a loss of mitochondrial membrane potential, translocation of Bax to mitochondria, release of cytochrome c, Smac/DIABLO and apoptosis inducing factor, suggesting the activation of the intrinsic apoptotic pathway [38]. Additionally, WSF treated HL-60 cells showed an over-expression of TNF receptor-1 and death receptor-4 with associated caspase-8 activation, suggesting the activation of the extrinsic apoptotic pathway [38]. Our 24 h results showed that W_RE_ increased PBMC caspase -8, -9 and -3/7 activities at different concentrations, suggesting the activation of extrinsic and intrinsic apoptotic pathways. In the same time period, W_RE_ increased THP-1 caspase -9 and -3/7 activities, suggesting initiation of apoptosis through the intrinsic pathway. At 72 h, W_RE_ (0.05–0.1 mg/ml) increased caspase (-8, -9, -3/7) activities, suggesting an increased initiation of PBMC apoptotic cell death. However, 0.4 mg/ml W_RE_ decreased initiator caspase (-8, -9) activities, suggesting a decreased initiation of PBMC apoptotic cell death. In THP-1 cells, 0.1–0.2 mg/ml W_RE_ (72 h) increased caspase (-8, -9, -3/7) activities, suggesting initiation of apoptosis through the intrinsic and extrinsic pathways. However, at 0.05 and 0.4 mg/ml W_RE_ (72 h), THP-1 caspase (-8, -9, -3/7) activities were decreased, suggesting a decrease in THP-1 apoptosis. Previous studies have indicated that *W. somnifera* may activate the extrinsic and intrinsic apoptotic pathways [38], therefore our results prove to be consistent with other studies.

Increased caspase-3 activity, proteasome activity and E3 ubiquitin-conjugating enzyme expression is associated with increased proteolysis [51]. Therefore the ability of W_RE_ (0.4 mg/ml, 72 h) to down regulate caspase activity in PBMC’s and THP-1 cells may decrease proteolysis and the progression of cancer cachexia.

A successful anti-cancer drug should kill or incapacitate cancer cells without causing excessive damage to normal cells [39]. The potential of W_RE_ to regulate PBMC apoptosis while increasing cancerous THP-1 cell apoptosis may be beneficial to cancer patients by preventing excessive cancerous cell growth while minimally effecting healthy PBMC’s.

## Conclusion

The cachectic syndrome decreases the quality of life of patients, the responsiveness to chemotherapy and leads to 20–25% of cancer deaths [3]. Our results show that W_RE_ increased oxidant scavenging activity, modulated GSH and pro-inflammatory cytokine levels and regulated caspase activity in normal PBMC’s and THP-1 cells. The discovery of a medicinal plant capable of decreasing the levels of pro-inflammatory cytokines may decrease tissue wasting. In this study, the root extract of *W. somnifera* has shown promising results in modulating the production of cytokines associated with cancer cachexia. The ability of W_RE_ to decrease pro-inflammatory cytokine levels and increase cancerous cell death may decrease the development and progression of cancer and cachexia. W_RE_ may therefore be effective in cancer cachexia.

## Additional file

Additional file 1: Assay data from root extract of *W. Somnifera*. (XLSX 288 kb)

## Abbreviations

ATP: Adenosine triphosphate
BHT: butylated hydroxytoluene
DNA: Deoxyribonucleic acid
DPPH: 2, 2-diphenyl-1 picrylhydrazyl
ELISA: enzyme-linked immunosorbant assay
FA’s: fatty acids
FCS: Foetal calf serum
GSH: Gluthatione
h: Hours
HL-60: Human promyelocytic leukemia
HSL: hormone sensitive lipase
IL: interleukin
LPL: lipoprotein lipase
LPS: lipopolysaccharide
min: Minute
NF-κB: Nuclear factor kappa B
NO: nitric oxide
PARP: poly (ADP-ribose) polymerase
PBMC’s: peripheral blood mononuclear cells
PBS: phosphate buffered saline
PSF: Pen/Strep Amphotericin B
RLU: Relative light units
ROS: reactive oxygen species
RT: room temperature
SA: South Africa
TAG: triacylglycerol
THP-1: leukemic cell line
TNF-α: tumour necrosis factor-α
*W. somnifera*: *Withania somnifera*
W_RE_: *W. somnifera* aqueous root extract
WSF: *W. somnifera* formulation
WST-1: 4-[3-(4-iodophenyl)-2-(4-nitrophenyl)-2H-5-tetrazolio]-1,3-benzene disulfonate
